# Intraocular foreign bodies extracted by pars plana vitrectomy: clinical characteristics, management, outcomes and prognostic factors

**DOI:** 10.1186/s12886-015-0128-6

**Published:** 2015-11-02

**Authors:** Simona Delia Nicoară, Iulian Irimescu, Tudor Călinici, Cristina Cristian

**Affiliations:** Department of Ophthalmology, “Iuliu Hațieganu” University of Medicine and Pharmacy, 8, V. Babeș str, 400012 Cluj-Napoca, Romania; Department of Neuroscience, “Iuliu Hațieganu” University of Medicine and Pharmacy, Cluj-Napoca, Romania; Department of Medical Informatics and Biostatistics, “Iuliu Hațieganu” University of Medicine and Pharmacy, Cluj-Napoca, Romania

**Keywords:** Intraocular foreign body, Pars plana vitrectomy, Ocular trauma

## Abstract

**Background:**

Intraocular foreign bodies (IOFBs) are an important cause of visual loss within the group of working age population. We aim to present the clinical features and the algorithm according to which we manage the foreign bodies that are located in the posterior segment of the eye. We define the outcomes and the prognostic factors that influenced the final visual acuity and globe survival in patients with IOFBs that we extracted by pars plana vitrectomy (PPV) over a 5-year period.

**Methods:**

We reviewed the medical records of all the cases with IOFBs that we removed by PPV, over 5 years (2009–2013). We extracted the following parameters: age, gender, wound anatomy, IOFB characteristics, ocular lesions, initial and final visual acuities. We used the program SPSS version 20.0.0. for the statistical analysis of our data.

**Results:**

During 5 years, we treated 21 IOFBs by PPV, representing 12.20 % of all the open globe injuries. All the patients were males with the median age of 36 years. The foreign body was located in the vitreous - 11 cases (52.38 %), retina - seven cases (33.33 %) and perforating - three cases (14.28 %). Retinal detachment (RD) at presentation was identified in eight cases (38.09 %) and endophthalmitis, in six cases (28.57 %). The visual outcome was significantly worse in patients with RD at presentation (*p* = 0.012) and with IOFBs larger than 3 mm (*p* = 0.042). Endophthalmitis did not influence the visual outcome.

**Conclusions:**

The worse prognostic factors were: RD at presentation and large foreign body.

**Trial registration number:**

IRCT2015040418966N3 / Apr. 9/2015

## Background

Ocular trauma is an important cause of visual morbidity and blindness, mainly in the group of working- age population [1, 2]. In this context, it was proved that intraocular foreign bodies (IOFBs) can lead to increased ocular morbidity [3, 4]. The development of vitreo-retinal surgery techniques and instrumentation has allowed to optimize the management of these complicated cases [5]. In this study we analyze all the consecutive cases with open-globe injuries and IOFBs that we treated by pars plana vitrectomy (PPV), in order to define the factors that affected the final visual acuity and globe survival.

## Methods

### Setting

This study was undertaken in the Department of Ophthalmology, belonging to the “Iuliu Hațieganu” University of Medicine and Pharmacy from Cluj-Napoca, Romania. The patients were enrolled in the study after they signed the informed consent form. The Ethics Committee of the “Iuliu Hațieganu” University of Medicine and Pharmacy approved the study.

### Study sample

All the consecutive patients who were diagnosed with IOFBs that were treated by PPV between January 1st 2009 and December 31^st^ 2013 were included in the study. The sampling method is longitudinal retrospective.

### Medical intervention

The medical intervention consisted in PPV and IOFB removal with the intraocular magnet. Other surgical gestures were associated, according to the situation: lens extraction (via the anterior chamber or by pars plana), intraocular lens (IOL) implantation, repair of retinal break/detachment.

### Statistical analysis

The statistical analysis of our data was performed with the program SPSS, version 20.0.0 (Chicago, Illinois, USA). There were calculated frequencies for the following parameters: age, gender, wound anatomy, IOFB characteristics (chemical nature, size, location), the association of other ocular lesions (direct traumatic cataract, retinal break/detachment, ocular tissue prolapse, endophthalmitis), initial and final visual acuity (VA). Outcome was evaluated according to the final VA: below 0.1 or equal to or more than 0.1. Statistical analyses were used to compare treatment outcomes among the study groups. Chi–square correlation was used to calculate the correlations between the categorical variables. Value <0.05 was considered statistically significant. In case of unequal data distribution, the *p* value was given by Fisher’s Exact test.

## Results and discussion

### Epidemiological data

Between January 1^st^ 2009 and December 31^st^ 2013, we carried out PPV with the aim to extract 21 IOFBs, representing 12.20 % of the 172 open globe injuries that were treated in our hospital during the same period. The demographic data of our patients are illustrated in Table 1.

**Table 1:** Demographic data of the cases with IOFB extracted by PPV

Age	
Median (range, SD)	36 (16–62, 14.77)
Number of hospital days	
Median (range, SD)	9.04 (2–25, 6)
Gender	
Male (%)	21 (100 %)
Female (%)	0
Eye	
Right (%)	5 (23.81 %)
Left (%)	16 (76.19 %)
Eye protection	
Yes (%)	0
No (%)	21 (100 %)

*SD* standard deviation

Different studies reported the presence of IOFBs in 10 to 14 % of open globe injuries [6–10]. This study refers strictly to the IOFBs located in the posterior segment that were extracted by PPV. They were present in 21 of the 172 open globe injuries that we treated during the last 5 years (12.20 %). If the open globe injury is complicated by endophthalmitis, IOFBs can be as frequent as 53 % of cases [11]. On our series, IOFBs were associated in 57.14 % of traumatic endophthalmities during the same time frame (8 IOFBs in 14 traumatic endophthalmities). Of the above mentioned eight IOFBs, six were located in the posterior segment and are included in this study. Our data confirm the findings in the literature, that most patients are young (median age 36 years), males (100 %) and with good pre-injury visual acuities [11]. The typical mechanism of trauma was hammering and shaving metal, either at work or at home. None of our patients was wearing eye protection during the accident. Therefore, we emphasize that the use of protection should play a major role in preventing this disability affecting young people.

### Clinical overview of the cases

The data of the eye examination at presentation are illustrated in Table 2. The location of the IOFB in the posterior segment is illustrated in Table 3. The most important clinical data and the visual acuities before and after surgery are summarized in Table 4.

**Table 2:** Data of the objective eye examination at presentation in the IOFB patients

Clinical characteristic	Number of cases (%)
Wound anatomy (*n* = 21 )	
Cornea involved (%)	17 (80.95 %)
Central (%)	11 (52.38 %)
Peripheral (%)	6 (28.57 %)
Limbus involved (%)	1 (4.76 %)
Sclera involved (%)	4 (19.04 %)
Average length, mm (range, SD)	3 (0.5 – 12, 3.54)
Initial eye examination	
Hyphema	5 (23.80 %)
Affected iris	16 (76.19 %)
Direct cataract	13 (61.90 %)
Vitreous hemorrhage	21 (100.00 %)
Retinal detachment	8 (38.09 %)
Endophthalmitis	6 (28.57 %)

**Table 3:** IOFB location

IOFB location	Number of cases (%)
In the vitreous	11 (52.38 %)
In the retina	7 (33.33 %)
Perforating	3 (14.28 %)

**Table 4:** Summary of the patients with IOFBs extracted by PPV

Case	InitialVA	IOFB location	IOFB dimension (mm)	Retinal detachment	Endophthalmitis	Cataract	Final VA
1	HM^a^	Vitreous	2	No	Yes	No	NLP
2	HM	Vitreous	5	Yes	No	Yes	HM
3	HM	Vitreous	1	No	Yes	No	HM
4	0.01	Retina	1	No	No	Yes	0.7
5	0.02	Vitreous	1.5	No	Yes	Yes	0.3
6	0.01	Vitreous	3	No	No	Yes	0.1
7	HM	Vitreous	7	Yes	No	Yes	HM
8	NLP^b^	Perforating	11(CT)	Yes	No	Yes	NLP
9	HM	Vitreous	1.5	Yes	No	Yes	HM
10	HM	Retina	7	Yes	No	No	HM
11	HM	Retina	3	No	No	Yes	0.1
12	0.02	Vitreous	1	No	No	Yes	0.3
13	0.03	Retina	1	No	No	No	0.3
14	0.04	Vitreous	1	No	No	No	0.2
15	HM	Vitreous	2	No	Yes	Yes	0.6
16	NLP	Perforating	10 (CT)	Yes	No	No	NLP
17	LP^c^	Perforating	8 (CT)	Yes	No	No	HM
18	HM	Vitreous	5	No	Yes	Yes	0.1
19	HM	Retina	5	No	Yes	No	HM
20	0.6	Retina	2	No	No	Yes	0.8
21	NLP	Retina	12	Yes	No	Yes	NLP

^a^ *HM* hand motion, ^b^ *NLP* no light perception, ^c^ *LP* light perception

Retinal detachment (RD) has been reported to occur in up to 30 % of open-globe injuries and 6–36 % of those with posterior segment IOFBs [12]. Retinal detachment at the moment of the IOFB extraction was identified in eight cases on our series (38.09 %). As proved by our series, IOFBs are frequently associated with hyphema, cataract, vitreous hemorrhage (Table 2). The endophthalmitis risk in the IOFB cases varies between three and 30 %, according to the different studies [7, 12]. Endophthalmitis was present in 6 of the 21 IOFBs on our series (28.57 %).

### Diagnosis

Diagnostic imaging was performed preoperatively in all eyes. seven of the 21 eyes (33.33 %) underwent computerized tomography (CT), with thin (1 mm) sections through the head and orbits with axial and coronal planes. CT was sensitive in 100 % of cases. Echography was performed in all cases, but with a sensitivity of 90.47 % (positive in 19 of the 21 cases). All patients underwent orbital radiogram, which was 100 % sensitive, but had the disadvantage of localizing the foreign body imprecisely.

The ideal imaging method for the diagnosis of IOFBs is the CT. We could perform it in only 33.33 % of our cases, because of economic reasons. All patients underwent orbital radiograms, because it’s very accurate in diagnosing the presence of the IOFB (100 % sensitivity), completed with echography, for localizing the IOFB. However, echography was not 100 % sensitive, as it could not always indicate the nature of a reflective lesion.

### Treatment

**Fig. 1:**
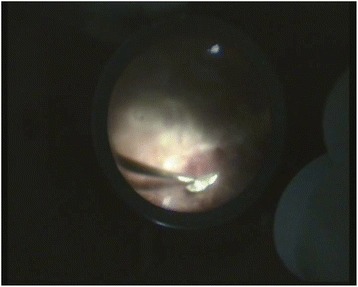
IOFB approach with the intraocular magnet

**Fig. 2:**
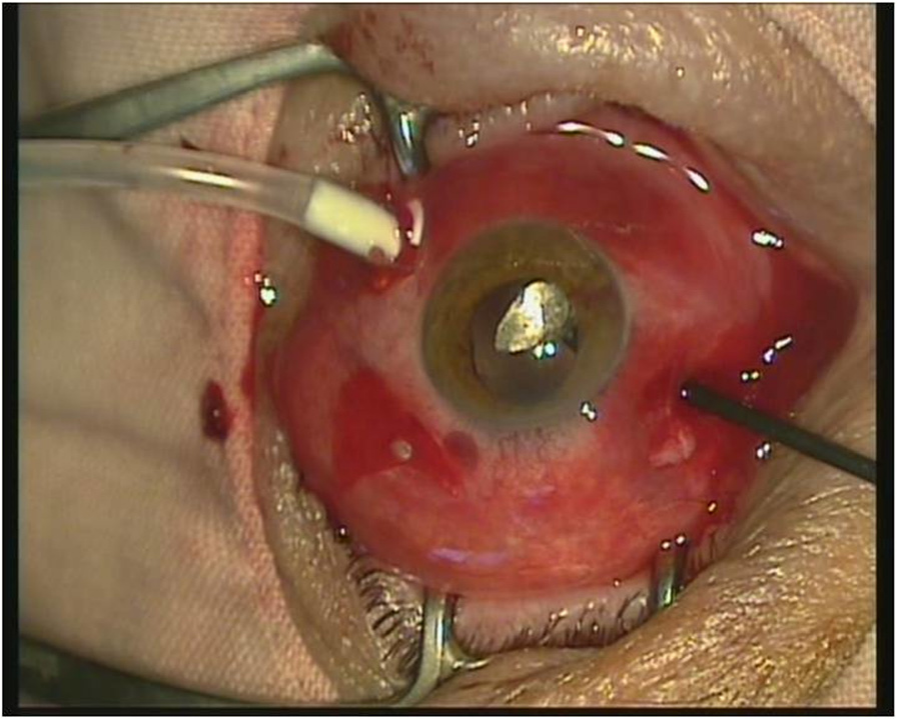
IOFB brought in the anterior segment with the intraocular magnet

PPV was carried out in all cases within 24 h from admission in our hospital, in 11 cases we used 20G vitrectomy and in ten cases, 25G vitrectomy. Of the 21 foreign bodies, three were perforating. The exit wounds were larger than the entrance ones and located as follows: transfoveal (case 8), nasally to the optic disc (case 16) and inferior, in the mid periphery of the retina (case 17). In all these three circumstances, the IOFBs were left in the orbit. Retinal detachment was associated in all the three cases and it was treated on the same session. The remaining 18 IOFBs were all metallic and magnetic. After having completed the vitrectomy and released the IOFB from the adherences with the vitreous and the retina, we enlarged one sclerotomy and extracted the IOFB with the intraocular magnet (Figs. 1 and 2).

**Fig. 3:**
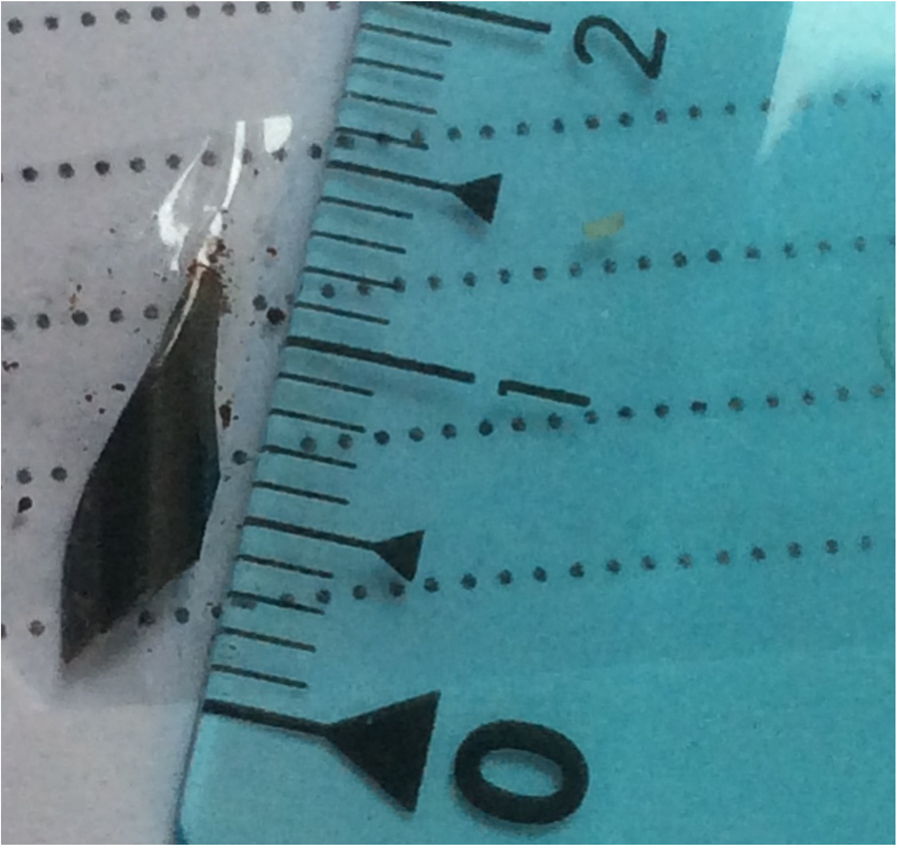
Large IOFB (12 mm)

**Fig. 4:**
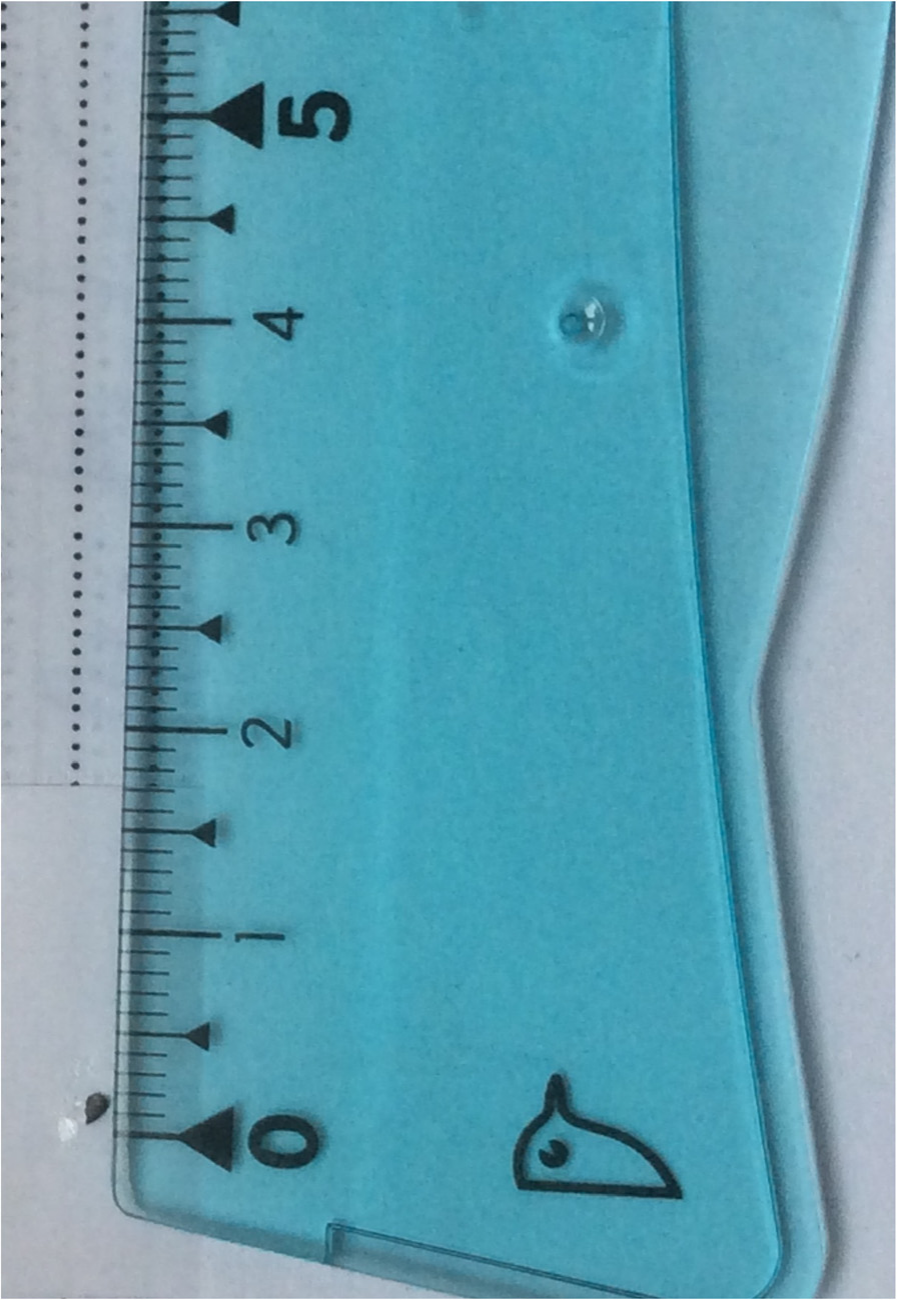
Small IOFB (2 mm)

We had one case with a 12 mm IOFB (Fig. 3), in which extraction was attempted with the extraocular magnet, in another service. In this case, we carried out PPV with the aim to dissect the adherences between the IOFB and the retina and vitreous. Then we extracted it with the extraocular magnet, via the entrance, large, corneal wound. At the opposite, Fig. 4 presents one of the smallest IOFBs in our series.

Retinal lesions, if present, were treated in all cases by endolaser photocoagulation around them and if retinal detachment was associated, silicone oil was injected after having reattached the retina. In the cases with direct cataract (13 cases), the lens was extracted at the same time with the primary repair, via the anterior chamber (7 cases) or by pars plana (6 cases). The implantation of an intraocular lens (IOL) was performed in the same session (2 cases) or later (11 cases). In the cases complicated with endophthalmitis, at the end of surgery we injected in the viteous cavity vancomycin 1.0 mg/0.1 cc, ceftazidime 2.2 mg/0.1 cc and dexamethasone 0.4 mg/0.1 cc.

### Rationale for using PPV for IOFB removal

Before the development of PPV, all the magnetic foreign bodies were extracted from the eye with the external magnet [5]. About 55–60 % of these eyes achieved visual acuities of 20/40 or better [13–16]. The drawback comes from the high risk of retinal detachment in eyes that develop vitreous organization and shortening of the pre-retinal fibrous bands. All the IOFBs on our series were metallic and magnetic. The severity of IOFBs is given by the risk of infection, toxic reactions and ocular lesions produced during their intraocular trajectory [11]. The argument in favor of PPV in the management of IOFBs is that removing the damaged vitreous decreases the risk of retinal detachment. Also, a fibrin capsule develops within hours around the foreign body, preventing its extraction with the external magnet. Moreover, during the application of the external magnet on the eye wall, significant traction may develop from the adherences between the IOFB and the retina, with high risk of iatrogenic retinal breaks. Also, if the foreign body is embedded in the retina, it cannot be extracted with the extraocular magnet. Furthermore, the IOFBs are often accompanied by vitreous hemorrhage and sometimes retinal breaks and detachment are identified. Some claim that if the IOFB is located on the peripheral retina, it may be removed by external magnet with scleral cut down, if minimal vitreous disruption is anticipated [11]. We do not support this approach, as it is associated with a high risk of vitreous hemorrhage and proliferative vitreo-retinopathy (PVR) [17]. We remove all the IOFBs located in the posterior segment by PPV. This attitude allows us to identify retinal lesions that the foreign body may have produced along its trajectory and that may have been missed without PPV, and to address them adequately. Also, the irrigation fluid through the vitreous cavity lessens the risk of endophthalmitis [11].

The use of PPV dramatically decreased the risk of retinal detachment in the period following IOFB extraction. Thus, in older studies, retinal detachment rates after primary surgery was up to 79 %, whereas in more recent ones, 11–23 % [18, 19].

On our series, retinal detachment occurred in 1 of the 13 IOFB cases without retinal detachment at presentation (7.69 %), a lower rate as compared to the literature, which may be explained by our relatively short follow up time. The average length of the follow up period in our series was 6 months. In all cases we considered as final, the VA measured at the last ophthalmological exam, which varied between 3 months and 1 year after the last surgery.

### Timing

It is agreed that IOFBs need to be removed, because of the risk of endophthalmitis (3–30 % of IOFBs) and toxic reactions [7, 12, 20, 21]. Timing is controversial. Previous studies showed that the delay in IOFBs removal is associated with a higher risk of endophthalmitis [22]. These observations are contradicted by the report of Coyler and colleagues who proved that the delay of combat-related IOFBs removal was not followed by endophthalmitis [23]. Despite this, most studies recommend the prompt removal of the IOFBs, within 24–48 h after trauma [16–18]. This is also our approach, but being a tertiary care center, sometimes older cases are referred to us. If endophthalmitis is associated, surgery is urgent. According to the literature, when endophthalmitis complicates the IOFB cases, it is identified preoperatively in about half of the cases and postoperatively, in the other half [24]. On our series, all the endophthalmitis cases were already present at the moment of IOFB extraction and in the postoperative period, no new case developed. The desideratum to remove IOFBs quickly is not always easily achieved, because the surgery must be performed by a trained surgeon and staff.

### Peculiarities of PPV in the removal of IOFBs

The surgical priorities are: the closure of the entry site, the removal of the IOFB and the prevention/treatment of endophthalmitis. Associated gestures, like cataract removal and IOL implantation come on the second place. PPV performed in the trauma setting is technically more demanding as in other circumstances. Visualization is difficult, because of the intraoperative bleeding, inflammation and other consequences of trauma, associated with the IOFBs. This is embarrassing, especially during the repair of the retinal detachment, when the lack of visibility sometimes prevents the finalization of surgery. The delay until the media clear up decreases the chances for vision recovery, as proliferative vitreo-retinopathy (PVR) may develop within days, with irreversible damage on the retina. On the other hand, continuing surgery under poor visualization conditions carries the risk of inducing iatrogenic lesions that aggravate the condition. These difficulties are proved by the bad outcome of all our IOFB cases with retinal detachment upon presentation, none of them recovered useful vision. Also, because of the penetrating eye lesion, PPV is not performed in a perfectly closed system, even if the suture preceded it. In consequence, the eye becomes hypotonic during surgery and visibility decreases, especially if the wound is large.

### Outcome

In order to define the outcome of our patients, we considered as “useful vision”, a visual acuity equal to or higher than 0.1. We use the decimal system to express the visual acuity. Overall, 10 of the 21 patients with IOFBs recovered useful vision (47.61 %).

None of the three patients with perforating IOFBs recovered useful vision. From the 11 cases with IOFBs located in the vitreous, six cases recovered visual acuities equal to or higher than 0.1 (54.54 %) and from the seven cases with intraretinal foreign bodies, in four cases the final VA was equal to or higher than 0.1 (57.14 %). The difference is not statistically significant (*p* > 0.05).

Retinal detachment at presentation was identified in eight cases and none of them recovered useful vision. From the 13 cases without RD at presentation, in ten cases the VA was 0.1 or higher (76.92 %). The difference is statistically significant (*p* = 0.012).

Within the endophthalmitis subgroup (6 cases), three patients recovered VA ≥ 0.1 (50 %) and within the non-endophthalmitis subgroup (15 cases), seven patients recovered VA ≥ 0.1 (46.66 %). The difference is not statistically significant (*p* > 0.05).

The dimensions of the IOFBs varied between 1 mm and 12 mm (Table 4). Because the IOFBs that perforated the eyes were left in the orbits, we noted their dimensions according to the data of the CT examination. In order to find out if the IOFB dimension influenced the outcome of VA, we divided the patients in two subgroups: with IOFBs less/equal to 3 mm (14 cases), and larger than 3 mm (7 cases). Within the subgroup of IOFBs less/equal to 3 mm, ten of the 14 patients recovered useful vision (71.42 %). None of the patients with IOFBs larger than 3 mm (7 cases) recovered useful vision. The difference between the 2 subgroups in terms of VA recovery is statistically significant (*p* = 0.043).

In the postoperative period, retinal detachment developed in one case (case 3, Table 4) that had endophthalmitis upon presentation. Despite surgery, retina detached and visual acuity remained hand motion.

### Prognostic factors

The factors that may influence the final visual outcome are listed in Table 5. The worse prognostic factors according to the interpretation of our results were: the large IOFB and the RD at presentation.

**Table 5:** Factors that may influence the final visual outcome

Factor		Number of eyes (%)	Number of eyes with final VA ≥ 0.1 (%)	Number of eyes with final VA < 0.1 (%)	*p*-value
Size of IOFB	≤3 mm	14 (66.66 %)	10 (71.42 %)	4 (28.58 %)	0.043*
	>3 mm	7 (33.34 %)	0 (0 %)	7 (100 %)	
RD	Yes	8 (38.09 %)	0 (0 %)	8 (100 %)	0.012*
	No	13 (61.91 %)	10 (76.92 %)	3 (23.08 %)	
Endophthalmitis	Yes	6 (28.57 %)	3 (50 %)	3 (50 %)	0.214
	No	15 (71.43 %)	7 (46.66 %)	8 (53.34 %)	
Location of IOFB	Retinal	7 (33.33 %)	4 (57.14 %)	3 (42.86 %)	0.914
	Vitreal	11 (52.38 %)	6 (54.54 %)	5 (45.46 %)	
Initial VA	≥0.1	4 (19.95 %)	4 (100 %)	0 (0 %)	0.760
	<0.1	17 (80.95 %)	6 (35.29 %)	11 (64.71 %)	
Lens injury	Yes	13 (61.90 %)	8 (61.53 %)	5 (38.47 %)	0.214
	No	8 (38.10 %)	2 (25 %)	6 (75 %)	
Iris injury	Yes	14 (66.66 %)	8 (57.14 %)	6 (42.86 %)	0.193
	No	7 (33.34 %)	2 (28.57 %)	5 (71.43 %)	
Hyphema	Yes	4 (19.95 %)	0 (0 %)	4 (100 %)	0.155
	No	17 (80.95 %)	10 (58.82 %)	7 (41.18 %)	

The association of endophthalmitis did not worsen the prognosis of the IOFB cases.

The intraretinal location of the foreign body was not associated with a worse visual outcome as compared to the vitreal location, if RD was not associated upon presentation. Endolaser photocoagulation of the retinal lesion at the moment of vitrectomy prevented further development of RD.

According to literature, better presenting visual acuity (VA) is considered a prognostic factor for a better visual outcome [2, 7, 9, 18]. Initial VA was ≥ 0.1 in four of our 21 cases (19.95 %) and all of them had final VA ≥ 0.1 (100 %). From the 17 cases with VA at presentation < 0.1, only 6 (35.29 %) reached final VA ≥ 0.1. However, this difference is not statistically significant (*p* = 0.760). Therefore, better presenting VA was not a prognostic factor for a better visual outcome in our series.

Statistical tests proved that the lesions of the anterior segment (lens, iris, hyphema) were not associated with worse visual outcome (Table 5). The recovery of VA depended mainly on the condition of the retina.

Even with the vitrectomy instrumentation and controlled removal of the IOFB, only about 55–60 % of cases achieve final VA of 20/40 or better [5]. On our series, ten cases had final VA of 0.1 or better (47.61 %), but only three cases (14.28 %) reached final VA better than 20/40. The poorest prognostic associations of IOFBs quoted in the literature are: endophthalmitis, retinal detachment, PVR [11]. On our series, we identified two factors associated with a worse outcome: retinal detachment at presentation and large foreign body. The two factors are directly related, in all the cases with RD at presentation, the dimension of the IOFB was over 5 mm. Endophthalmitis was not demonstrated to be a worse prognostic factor on our series. The risk of PVR is increased by the extensive retinal and choroidal lesions, vitreous hemorrhage and posterior location of the wound [25, 26]. On our series, PVR compromised the recovery of the visual function in nine cases (42.85 %), of which eight have had RD upon presentation and one developed RD after the IOFB extraction.

## Conclusions

We identified two factors that were significantly associated with a worse outcome in our series: retinal detachment upon presentation and large IOFB. The extensive retinal and choroidal lesions produced by the large IOFBs induced the development of PVR that eventually led to the loss of vision. Given the poor outcomes associated especially with IOFBs larger than 3 mm, eye protection should play a major role in preventing this disability affecting young people.

## Abbreviations

CT: Computer tomography
HM: Hand motion
IOFBs: Intraocular foreign bodies
IOL: Intraocular lens
LP: Light perception
NLP: No light perception
PPV: Pars plana vitrectomy
PVR: Proliferative vitreoretinopathy
RD: Retinal detachment
SD: Standard deviation
VA: Visual acuity
